# Association Between Hepatitis C Virus Infection and Coronary Artery Disease: A Systematic Review and Meta-Analysis

**DOI:** 10.14740/cr2280

**Published:** 2026-10-24

**Authors:** Ali Alhashli

**Affiliations:** Department of Internal Medicine, King Faisal Specialist Hospital and Research Centre, Riyadh, Saudi Arabia. Email: hashili_94@live.com

**Keywords:** Cardiovascular disease, Coronary artery disease, Meta-analysis, Systematic review, HIV co-infection, Direct-acting antivirals, Hepatitis C virus

## Abstract

**Background:**

Hepatitis C virus (HCV) infection has been associated with cardiovascular disease, but its relationship with coronary artery disease (CAD) remains uncertain.

**Methods:**

This systematic review included observational studies reporting adjusted effect estimates for CAD outcomes among HCV-infected and HCV-uninfected adults. Adjusted hazard ratios, risk ratios, and odds ratios were combined on the logarithmic scale using a random-effects model and are presented as a summary relative association estimate with 95% confidence intervals (CIs). Prespecified subgroup analyses were performed by study design, human immunodeficiency virus (HIV) co-infection status, and CAD outcome type; meta-regression evaluated follow-up duration, mean age, and the proportion of male participants.

**Results:**

Twenty-one studies were included, representing diverse populations including veterans, people living with HIV, dialysis patients, and community-based cohorts. Fifteen studies contributed adjusted estimates to the quantitative synthesis. HCV infection was associated with a modest increase in the pooled relative estimate for CAD (summary estimate 1.21; 95% CI, 1.05–1.40; P = 0.007), with substantial heterogeneity (I^2^ = 72.1%). The association was statistically significant in cohort studies and in both HIV-specific and non-HIV-specific subgroups, although the HIV-specific subgroup was based on four studies. The myocardial infarction (MI)-specific subgroup was not statistically significant, whereas studies using broader CAD outcomes showed a significant association.

**Conclusions:**

Chronic HCV infection was associated with a modest increase in the pooled relative estimate for CAD. However, substantial heterogeneity, differences in endpoint definitions and populations, residual confounding, and the combination of different effect measures limit causal interpretation and make the clinical importance uncertain. The nonsignificant MI-specific analysis further supports a cautious interpretation. These findings do not establish a cardiovascular benefit of antiviral therapy; prospective studies with standardized CAD endpoints and detailed data on renal disease, antiviral exposure, and lipid-lowering therapy are needed.

## Introduction

Hepatitis C virus (HCV) infection is a significant global burden of public health, with approximately 58 million people infected worldwide at the start of 2020, and the chronic form of this infection causes progressive hepatic fibrosis, cirrhosis, and hepatocellular carcinoma in a significant percentage of patients [1, 2]. In addition to its long-known hepatic effects, HCV is now being acknowledged as a systemic illness with significant extrahepatic effects including metabolic, renal, hematological, and cardiovascular sequelae [3, 4]. Specifically, chronic HCV infection and coronary artery disease (CAD) have received significant scientific interest due to the high incidence of cardiovascular diseases in the world and how HCV-mediated pathobiological effects may accelerate the atherosclerosis process [5].

There are several biologically plausible mechanisms through which HCV could be associated with accelerated atherogenesis. Chronic HCV infection promotes a pro-inflammatory state characterized by activation of innate immune pathways, upregulation of pro-inflammatory cytokines, and endothelial dysfunction [6, 7]. HCV-associated hepatic steatosis and insulin resistance may also increase cardiometabolic risk [8]. HCV RNA has been detected in carotid atherosclerotic plaques, suggesting a possible direct viral contribution to arterial-wall pathology [9]. In contrast, chronic HCV infection is often accompanied by lower total and low-density lipoprotein (LDL) cholesterol concentrations, a lipid pattern that would ordinarily be expected to reduce atherosclerotic risk; lipid concentrations may rise after successful viral clearance [10]. Thus, the apparently favorable lipid profile of active HCV infection coexists with inflammatory, endothelial, and metabolic abnormalities that may increase vascular risk. This biological tension may partly explain the inconsistent epidemiological findings.

Early systematic reviews reported inconsistent findings and highlighted substantial clinical and methodological heterogeneity [11]. Subsequent meta-analyses found positive associations across broader cardiovascular outcomes. Ambrosino et al reported an increased pooled estimate for CAD and cerebrovascular disease but included both adjusted and unadjusted effect sizes and did not stratify by human immunodeficiency virus (HIV) status [12]. Petta et al synthesized 22 studies and separately evaluated cardiovascular mortality, carotid atherosclerosis, and cerebrocardiovascular events [13]. Lee et al included 36 longitudinal studies, reported a pooled risk ratio of 1.28 (95% confidence intervals (CI), 1.18–1.39), and presented separate analyses for composite cardiovascular events, myocardial infarction (MI), and stroke [14].

Accordingly, the present review should be viewed as an updated, CAD-focused synthesis rather than the first outcome-stratified meta-analysis. Its principal additional focus is the comparison of HIV-specific and non-HIV-specific populations, together with meta-regression of study-level follow-up duration, mean age, and sex composition. Nevertheless, variation in CAD definitions, treatment eras, comorbidity profiles, and effect measures remains an important methodological challenge.

The objective of this systematic review and meta-analysis was to update the evidence on the association between chronic HCV infection and CAD, with subgroup analyses by study design, HIV co-infection status, and CAD outcome type, and meta-regression of continuous study-level moderators. Given the observational design and anticipated heterogeneity, the analysis was intended to estimate association rather than causality or treatment effects.

## Materials and Methods

This systematic review and meta-analysis was conducted and reported in accordance with the Preferred Reporting Items for Systematic Reviews and Meta-Analyses (PRISMA) 2020 statement [15].

### Search and selection

An electronic database search was performed on four databases (PubMed, Scopus, Web of Science and Cochrane Library). The complete search strategy for each database is provided here (Supplementary Material 1, cr.elmerpub.com). There were no language or date constraints. Included studies and relevant reviews were screened and used to generate reference lists of other eligible studies.

### Eligibility criteria

Studies were eligible if they: (1) used an observational cohort, case-control, or cross-sectional design; (2) included adults aged 18 years or older with chronic HCV infection identified by serology, serum HCV RNA, or diagnostic codes; (3) reported a coronary outcome, including MI, acute coronary syndrome (ACS), incident CAD/coronary heart disease (CHD), or cardiovascular mortality; and (4) reported an adjusted odds ratio (OR), risk ratio (RR), or hazard ratio (HR), or provided data suitable for qualitative synthesis. Cardiovascular mortality was retained as an eligible outcome in the original review framework, although it is not clinically equivalent to incident CAD or MI; this distinction is addressed in the interpretation and limitations.

Studies were excluded if they were limited to prevalent CAD at baseline; were reviews, case reports, editorials, or conference abstracts with no full-text information; or reported non-coronary cardiovascular outcomes only (e.g., stroke, peripheral vascular disease) without a discrete CAD outcome.

### Screening and data extraction

Selection and screening were conducted by two independent reviewers, who screened titles and abstracts; full-text screening of potentially eligible articles was then performed and resolved by consensus. Two independent researchers extracted the data using a standardized form, which recorded: first author, year, country, study design, time frame, study population, sample size, HCV diagnosis method, CAD outcome definition and adjusted effect estimate with 95% CI and primary study conclusion (Table 1) [16–36]. When they were reported, baseline demographic and clinical data of HCV-infected and uninfected groups were extracted separately (Table 2) [16–36].

**Table 1:** Summary Characteristics for the Included Studies

Study	Study design	Country	Time frame	Total sample size	Type of population	HCV diagnosis	CAD outcome definition	Adjusted covariates	Follow-up, years	Conclusion
Arcari et al, 2006 [22]	Case-control study	USA	1991–2000	582	Men aged 30–50 years on active duty in the US Army	HCV-specific antibodies	MI based on ICD-9	Age, race, education, and marital status	-	Among active-duty military personnel with a high prevalence of HCV infection, no association has been observed between HCV infection and CHD.
Bedimo et al, 2010 [28]	Cohort study	USA	1996–2004	19,424	HIV-infected US veterans	Diagnostic codes (ICD-9) and HCV antibody tests	MI based on ICD-9	HTN, age, type 2 diabetes, and tobacco use	3.93	In the HAART era, HCV coinfection was associated with increased CVD risk and a trend toward higher AMI risk among HIV-infected individuals.
Butt et al, 2007 [33]	Cohort study	USA	1999–2003	253,942	US veterans between the ages of 20 and 90	One inpatient or two outpatient ICD-9 codes	CAD based on ICD-9	Age, race/ethnicity, sex, drug use, and alcohol use	—	HCV-infected individuals commonly present with multiple medical, psychiatric, and substance use comorbidities; however, they show lower odds of CAD and stroke. Psychiatric conditions are partly linked to substance use, and a comprehensive initial evaluation is recommended, while underlying mechanisms remain unclear.
Butt et al, 2009 [27]	Cohort study	USA	2001–2006	171,665	National cohort of HCV-infected veterans	HCV antibody or a positive qualitative/quantitative HCV RNA test	Incident CAD (≥ 1 inpatient or 2 outpatient ICD-9 codes for MI, CHF, CABG, or PTCA)	Age, race, sex, HTN, diabetes, hyperlipidemia, and COPD	—	HCV-infected individuals are typically younger with lower lipid levels and less HTN; however, HCV infection remains associated with an increased risk of CAD after adjustment for conventional risk factors.
Butt et al, 2017 [20]	Cohort study, PSM	USA	2001–2015	171,726	Male veterans	HCV antibody or HCV RNA test	Incident AMI (≥ 1 inpatient or 2 outpatient ICD-9 codes)	Age, race, BMI, and receipt of lipid-lowering agents	6.79	HCV-positive men have an increased risk of AMI at higher TC and LDL levels, particularly at younger ages; lipid-lowering therapy reduces this risk, with greater benefit observed compared to HCV-negative men.
Campbell et al, 2012 [21]	Cohort study	UK	January 2004 to December 2009	7,828	HIV-positive patients (≥ 18 years)	HCV antibody status	CHD events (MI, PTCA, CABG) from records and elevated troponin	Age, ethnicity, HIV RNA, eGFR, total/HDL cholesterol ratio, and tenofovir use	3.8	The incidence of CHD was low in this ethnically diverse cohort; impaired renal function identified individuals at higher risk, warranting prioritization of traditional risk factor management.
D:A:D study, 2010 [30]	Cohort study	Europe, USA, and Australia	December 1999 to February 2007	33,347	HIV-infected individuals	HCV serology (antibodies) and assessments of HCV load (RNA	Adjudicated MI (definite/possible) according to WHO MONICA criteria	Sex, age, HIV transmission risk, ethnicity, prior AIDS, smoking, family history of CVD, BMI, cumulative NNRTI/PI/NRTI exposure, prior CVD, cohort, incident DM, calendar year, latest CD4^+^ count, HIV RNA, TC, HDL-C, log-TG, HTN, and lipodystrophy	4.7	No association was observed between HBV or HCV coinfection and myocardial infarction among individuals with HIV.
Enger et al, 2014 [16]	Cohort study, PSM	USA	January 2000 to September 2006	90,931	Patients with HCV or cirrhosis	ICD-9-CM diagnosis codes	New-onset arterial thromboembolic events (MI, UA) using ICD-9-CM codes	Age, gender, HTN, past and recent corticosteroid use	7	Patients with HCV and cirrhosis from various etiologies have an increased risk of thromboembolic events, which should be considered in the clinical management of liver disease.
Forde et al, 2012 [19]	Cohort study, PSM	UK	February 1996 to November 2008	76,477	Adults (≥ 18 years) in UK general practices	Read diagnostic codes or free text comments	First occurrence of an acute MI using Read codes	Age, sex, comorbidities (HTN, diabetes, hyperlipidemia, family history of CVD and smoking), aspirin use, BMI, and tobacco use	3.2	HCV infection was not linked to an increased risk of incident MI.
Freiberg et al, 2011 [17]	Cohort study	USA	January 2000 to July 2007	6,154	Veterans with and without HIV and HCV	Positive HCV antibody test or ICD-9 codes	Incident AMI and UA (CHD) via ICD-9 codes 410–411	Age, race/ethnicity, education, BMI, HTN, DM, smoking, hypercholesterolemia, cocaine abuse/dependence, and alcohol abuse/dependence	7.3	HIV-positive individuals coinfected with HCV have a higher risk of CHD compared with those with HIV alone or neither infection.
Freiberg et al, 2013 [25]	Cohort study	USA	April 2003 to December 2009	82,459	HIV-positive and matched uninfected veterans	Positive HCV antibody test or ICD-9 codes	Adjudicated incident AMI using VA, Medicare, and death certificate data	Age, sex, race/ethnicity, HTN, DM, LDL-C, HDL-C, TG, smoking, statin use, HCV, eGFR, Hb, BMI, cocaine abuse/dependence, and alcohol abuse/dependence	5.9	HIV infection is associated with a 50% higher risk of AMI independent of established risk factors.
Hsu et al, 2014 [36]	Cohort study, PSM	Taiwan	2003–2011	7,055	Diabetic patients (ages 20–70)	Specific diagnosis code (ICD-9-CM) and serum antibody measurements	ACS hospitalizations with primary ICD-9-CM codes 410-414	HTN, dyslipidemia, COPD, PAOD, diabetes medication profile, aspirin, NSAIDs, ACEI/ARB, and statin use	3.8	Antiviral therapy for HCV infection is associated with improved renal and cardiovascular outcomes in patients with diabetes.
Kalantar-Zadeh et al, 2007 [29]	Cohort study	USA	July 2001 to June 2004	13,664	Maintenance hemodialysis patients	Third-generation HCV antibody enzyme immunoassay	Cardiovascular mortality	Age, sex, race/ethnicity, DM, smoking history, dialysis vintage, insurance, marital status, clinic SMR, Kt/V, dialysis catheter, residual renal function, and baseline comorbidities (IHD, CHF, prior cardiac arrest, prior MI, pericarditis, dysrhythmia, cerebrovascular disease, PVD, COPD, cancer)	3	In maintenance hemodialysis patients, HCV infection is associated with distinct demographic, clinical, and laboratory features, longer dialysis duration, and increased mortality; enhanced prevention and treatment may improve outcomes.
Lang et al, 2022 [32]	Cohort study	USA and Canada	January 2000 to December 2017	23,361	People with HIV (ages 40–79) who had initiated ART	Positive HCV antibody test, detectable RNA, or genotype result	Adjudicated T1MI event	Age, sex, race/ethnicity, HBV, DM, HTN, CKD, alcohol abuse/dependence, smoking, IDU history, statin use, HIV viral load, CD4 count, and PI use	3.9	HCV coinfection was not significantly associated with T1MI risk; however, the age-related increase in risk was greater among those with HCV, suggesting its relevance in cardiovascular risk assessment in aging people with HIV.
Lin et al, 2014 [18]	Cross-sectional study	Taiwan	December 2010	5,015	Community health examination participants (> 40 years)	Immunoassay for anti-HCV antibody	Ischemic ECG findings via Minnesota Code criteria	Sex, age, BW, waist, SBP, DBP, creatinine, uric acid, fasting glucose, TC, HDL-C, LDL-C, TG, HTN history, DM history, smoking, MetS, and HBV	—	HCV infection was strongly associated with ischemic ECG abnormalities in this community-based cohort and may represent a nontraditional risk factor for CAD.
Pothineni et al, 2014 [24]	Cohort study	USA	January 2001 to December 2013	16,233	Patients from a university-based electronic medical records database	HCV antibody (ELISA) and HCV RNA (PCR)	Incident CHD events (CAD, stable/UA, or MI) using ICD-9 codes	Age, sex, HTN, diabetes and chronic obstructive pulmonary disease	12	HCV seropositivity is associated with increased CHD incidence, particularly in those with detectable HCV RNA compared to antibody-positive individuals with resolved infection; lipid profile appears to have limited value for cardiovascular risk stratification in this population.
Satapathy et al, 2013 [26]	Case-control study	USA	May 2002 to December 2008	126	HCV patients and matched controls undergoing coronary angiography	ICD-9 codes and documented positive anti-HCV antibody test	> 50% blockage in a proximal coronary artery on angiogram	Age, DM, smoking, and family history of CAD	—	HCV infection is associated with higher prevalence and severity of CAD on angiographic evaluation compared with matched controls, suggesting a potential role as a risk factor for CAD.
Tsai et al, 2015 [23]	Cohort study	Taiwan	1998–2008	69,915	HCV patients without antiviral treatment and non-HCV companions	ICD-9-CM codes	ACS based on hospitalizations	Age, sex, and comorbidities of HTN, diabetes, hyperlipidemia, stroke, COPD, heart failure, hepatitis B, and cirrhosis	5.85	HCV infection is associated with an increased risk of ACS compared with non-infected individuals, with the highest risk observed in middle-aged patients.
Vassalle et al, 2004 [31]	Case-control study	Italy	—	686	Patients with angiographically documented CAD and matched controls	Antibodies against HCV	Angiographic documentation of CAD (> 50% stenosis)	Age, sex, smoking, HTN, DM, and dyslipidemia	—	HCV seropositivity may be considered a potential risk factor for the development of CAD, with possible additive effects alongside other pathogens; further studies are required to confirm its predictive and prognostic value.
Wang et al, 2023 [34]	Cohort study, PSM	USA	January 2008 to August 2015	197,484	Newly diagnosed HCV patients matched controls	ICD-9-CM diagnosis codes	CVD event defined by ICD-9-CM codes for CAD, CCV, or PVD	Age, sex, insurance plan type, alcohol abuse/dependence, drug abuse, HTN, dyslipidemia, DM, COPD, CKD, HIV, HBV, HAV, liver disease, and CVD-related medications (ACEIs, ARBs, alpha-beta blockers, beta-blockers, CCBs, diuretics, antihypertensives, cardiac glycosides, vasodilators, anticoagulants, antiarrhythmic drugs, lipid-lowering agents)	2.37	Chronic HCV infection is associated with a higher incidence of CVD, while antiviral treatment is linked to a reduced risk.
Williams-Nguyen et al, 2020 [35]	Cohort study	USA	1998–2016	23,407	People living with HIV	Detectable HCV RNA test	Centrally adjudicated MI is categorized as type 1 or type 2	Age, sex at birth, race/ethnicity, study site, men who had sex with men, ever smoking, history of injecting drug use, diabetes, statin use, HTN, antihypertensive medication use, hepatitis B virus infection, ART, CD4-positive cell count *nadir*, BMI, TC, HDL cholesterol, and TG levels	4.7	Chronic HCV infection at baseline was not associated with incident T1MI but was linked to type 2 MI.

HCV: hepatitis C virus; CAD: coronary artery disease; PSM: propensity score matching; HIV: human immunodeficiency virus; ELISA: enzyme-linked immunosorbent assay; PCR: polymerase chain reaction; ICD-9: International Classification of Diseases, Ninth Revision; MI: myocardial infarction; CHF: congestive heart failure; CABG: coronary artery bypass grafting; PTCA: percutaneous transluminal coronary angioplasty; AMI: acute myocardial infarction; CHD: coronary heart disease; WHO: World Health Organization; MONICA criteria: Monitoring Trends and Determinants in Cardiovascular Disease criteria; UA: unstable angina; VA: Veterans Affairs; ACS: acute coronary syndrome; CVD: cardiovascular disease; CCV: cerebrocardiovascular; PVD: peripheral vascular disease; eGFR: estimated glomerular filtration rate; HDL: high-density lipoprotein; AIDS: acquired immunodeficiency syndrome; BMI: body mass index; NNRTI: non-nucleoside reverse transcriptase inhibitor; PI: protease inhibitor; NRTI: nucleoside reverse transcriptase inhibitor; DM: diabetes mellitus; TC: total cholesterol; TG: triglycerides; HTN: hypertension; Hb: hemoglobin; COPD: chronic obstructive pulmonary disease; PAOD: peripheral arterial occlusive disease; NSAIDs: nonsteroidal anti-inflammatory drugs; ACEI: angiotensin-converting enzyme inhibitor; ARB: angiotensin receptor blocker; IHD: ischemic heart disease; SMR: standardized mortality ratio; Kt/V: dialyzer clearance of urea multiplied by dialysis time and divided by volume of distribution of urea; HBV: hepatitis B virus; CKD: chronic kidney disease; IDU: injection drug use; BW: body weight; SBP: systolic blood pressure; DBP: diastolic blood pressure; HAV: hepatitis A virus; CCBs: calcium channel blockers; ECG: electrocardiogram; HAART: highly active antiretroviral therapy; T1MI: acute myocardial infarction; ART: antiretroviral therapy.

**Table 2:** Baseline Characteristics of the Included Studies

Study	Groups	N	Age (years), mean (SD)	Sex (male), n (%)	BMI (kg/m^2^), mean (SD)	Smoking, n (%)	Cirrhosis, n (%)	HTN, n (%)	DM, n (%)	Hyperlipidemia, n (%)	TC (mg/dL), n (%)	TG (mg/dL), n (%)	LDL (mg/dL), n (%)	HDL (mg/dL), n (%)
Arcari et al, 2006 [22]	HCV	582	—	582 (100)	—	—	—	—	—	—	—	—	—	—
	Control		—		—	—	—	—	—	—	—	—	—	—
Bedimo et al, 2010 [28]	HCV	6,136	47 (7.31)	6,012 (97.98)	—	2,251 (36.69)	—	2,689 (43.82)	992 (16.17)	3,413 (55.63)	195.12 (59.63)	275.53 (274.15)	111.39 (44.25)	—
	Control	13,288	45.82 (11.25)	12,926 (97.28)	—	3,288 (24.74)	—	4,731 (35.60)	1,479 (11.13)	8,692 (65.41)	218.03 (70.60)	331.06 (352.99)	130.82 (45.18)	—
Butt et al, 2007 [33]	HCV	126,926	50.8	122,864 (96.8)	—	—	6,476 (5.1)	—	18,030 (14.2)	—	—	—	—	—
	Control	126,926	51.8	122,864 (96.8)	—	—	508 (0.4)	—	16,633 (13.1)	—	—	—	—	—
Butt et al, 2009 [27]	HCV	82,083	51.2 (7.3)	75,270 (91.7)	—	—	—	34,147 (41.6)	17,073 (20.8)	32,341 (39.4)	175 (40.8)	144 (119)	102 (36.8)	46 (16.8)
	Control	89,582	51.8 (7.8)	86,895 (97)	—	—	—	45,149 (50.4)	19,529 (21.8)	64,678 (72.2)	198 (41.9)	179 (151)	119 (38.2)	46 (15.2)
Butt et al, 2017 [20]	HCV	85,863	53.67 (6.67)	85,863 (100)	27.87 (4.67)	22,582 (26.3)	—	34,517 (40.2)	3,177 (3.7)	—	175 (36.7)	121.33 (64.49)	103.37 (32.84)	43.67 (13.34)
	Control	85,863	53.67 (6.67)	85,863 (100)	27.83 (4.60)	21,809 (25.4)	—	32,113 (37.4)	2,748 (3.2)	—	193.67 (36.7)	135.67 (77.84)	117.82 (32.84)	44.83 (12.97)
Campbell et al, 2012 [21]	HCV	7,828	—	—	—	—	—	—	—	—	—	—	—	—
	Control		—	—	—	—	—	—	—	—	—	—	—	—
D:A:D study, 2010 [30]	HCV	5,084	37 (5.93)	3,566 (70.1)	—	3,028 (59.6)	—	370 (7.3)	107 (2.1)	—	—	—	—	—
	Control	16,731	39 (9.64)	12,499 (74.7)	—	4,864 (29.1)	—	1,592 (9.5)	435 (2.6)	—	—	—	—	—
Enger et al, 2014 [16]	HCV	22,733	49 (9.7)	14,191 (62.4)	—	—	2,712 (11.9)	4,708 (20.7)	—	—	—	—	—	—
	Control	68,198	49 (9.8)	42,573 (62.4)	—	—	0 (0)	9,208 (13.5)	—	—	—	—	—	—
Forde et al, 2012 [19]	HCV	4,809	38.95 (11.20)	2,935 (61.03)	—	3,574 (74.32)	—	466 (9.69)	259 (5.39)	574 (11.94)	—	—	—	—
	Control	71,668	38.80 (11.16)	43,802 (61.12)	—	44,423 (61.98)	—	7,168 (10)	2,310 (3.22)	9,421 (13.15)	—	—	—	—
Freiberg et al, 2011 [17]	HCV	701	47.4 (5.6)	701 (100)	27.1 (5)	464 (66.2)	—	192 (27.4)	181 (25.8)	—	—	—	—	—
	Control	5,453	48.2 (9.9)	5,453 (100)	28.2 (5.3)	2,318 (42.5)	—	1,598 (29.3)	1,352 (24.8)	—	—	—	—	—
Freiberg et al, 2013 [25]	HCV	82,459	—	—	—	—	—	—	—	—	—	—	—	—
	Control		—	—	—	—	—	—	—	—	—	—	—	—
Hsu et al, 2014 [36]	HCV	1,411	55.01 (7.99)	918 (65.1)	—	—	322 (22.8)	606 (42.9)	1,411 (100)	175 (12.4)	—	—	—	—
	Control	5,644	54.93 (8.07)	3,680 (65.2)	—	—	1,289 (22.8)	2,444 (43.3)	5,644 (100)	718 (12.7)	—	—	—	—
Kalantar-Zadeh et al, 2007 [29]	HCV	1,590	54 (13)	1,034 (65)	25.2 (5.6)	127 (8)	—	—	604 (38)	—	—	—	—	—
	Control	12,074	61 (16)	6,399 (53)	26.4 (6.4)	483 (4)	—	—	5,796 (48)	—	—	—	—	—
Lang et al, 2022 [32]	HCV	4,677	46.67 (7.42)	3,621 (77.4)	25.17 (4.38)	3,539 (75.7)	—	1,314 (28.1)	381 (8.1)	—	164.33 (40.04)	—	89 (34.85)	42.67 (14.83)
	Control	18,684	45.33 (8.15)	15,670 (83.9)	25.93 (4.37)	10,552 (56.5)	—	4,881 (26.1)	1,304 (7)	—	184 (40.77)	—	106 (33.36)	41.67 (13.34)
Lin et al, 2014 [18]	HCV	593	51.48 (11.37)	240 (40.5)	—	153 (25.8)	—	68 (11.5)	48 (8.1)	—	187.22 (38.98)	111.72 (91.71)	119.66 (34.7)	58.76 (18.91)
	Control	4,422	62.49 (13.29)	2,017 (45.6)	—	1,027 (23.2)	—	570 (12.9)	277 (6.3)	—	186.52 (35.08)	104.68 (78.41)	119.48 (34.45)	59.87 (17.45)
Pothineni et al, 2014 [24]	HCV	1,434	48.6 (9.7)	817 (57)	—	—	—	443 (30.9)	234 (16.3)	—	156.5 (55.7)	138.8 (105)	87 (35.9)	43 (19.7)
	Control	14,799	53 (16.1)	7,992 (54)	—	—	—	1,805 (12.2)	755 (5.1)	—	185.8 (49.7)	163.3 (329.8)	107.7 (50.9)	47.3 (16.2)
Satapathy et al, 2013 [26]	HCV	63	60.9 (10.5)	41 (65.1)	32.2 (13.5)	29 (46)	—	52 (82.5)	28 (44.4)	33 (52.4)	147 (40)	125 (68)	91 (30)	42 (21)
	Control	63	60.8 (10.5)	41 (65.1)	29.8 (6.1)	26 (41.3)	—	51 (81)	26 (41.3)	43 (68.4)	144 (43)	102 (78)	88 (35)	42 (12)
Tsai et al, 2015 [23]	HCV	13,983	54.7 (15.8)	35,936	—	—	—	—	—	—	—	—	—	—
	Control	55,932	54.2 (16.1)		—	—	—	—	—	—	—	—	—	—
Vassalle et al, 2004 [31]	HCV	491	66 (11.08)	399 (81.2)	—	—	0 (0)	—	—	—	—	—	—	—
	Control	195	61 (11.6)	115 (58.9)	—	—	0 (0)	—	—	—	—	—	—	—
Wang et al, 2023 [34]	HCV	49,371	53 (8.15)	29,238 (59.22)	—	—	873 (1.77)	16,696 (33.82)	7,046 (14.27)	10,893 (22.06)	—	—	—	—
	Control	148,113	53 (8.15)	88,509 (59.76)	—	—	1994 (1.35)	51,251 (34.6)	21,294 (14.38)	31,871 (21.52)	—	—	—	—
Williams-Nguyen et al, 2020 [35]	HCV	2,280	—	1,779 (78)	25.6 (5)	873 (38.3)	—	370 (24.2)	163 (7.1)	—	159.5 (41)	157.2 (124)	—	43.5 (17)
	Control	21,127	—	17,117 (81)	25.9 (6)	5,160 (24.4)	—	3,450 (23.2)	1,030 (4.9)	—	174.9 (46)	170.2 (154)	—	42.1 (15)

HCV: hepatitis C virus; BMI: body mass index; HTN: hypertension; DM: diabetes mellitus; TC: total cholesterol; TG: triglycerides; LDL: low-density lipoprotein; HDL: high-density lipoprotein; SD: standard deviation; N: number.

### Risk of bias assessment

The Newcastle-Ottawa Scale (NOS) was used to assess the methodological quality of cohort and case-control studies based on the domains of selection, comparability, and outcome ascertainment with a maximum score of nine stars; those with a score of 6 or higher were considered to be of good quality, and those with a score of 4–5 to be of fair quality. The unpaired cross-sectional research was evaluated by an adapted NOS tool. The results of risk of bias are presented here (Supplementary Materials 2, 3, cr.elmerpub.com).

### Statistical analysis

Adjusted HRs, RRs, and ORs were entered on the logarithmic scale using their reported standard errors and pooled with a DerSimonian–Laird random-effects generic inverse-variance model. The exponentiated pooled value is labelled as an OR in the existing figures for consistency with the original analysis; throughout the revised text it is described as a summary relative association estimate because HRs, RRs, and ORs are not mathematically interchangeable. Studies without a separately extractable adjusted estimate for the prespecified HCV comparison and CAD outcome were included only in the qualitative synthesis. Statistical heterogeneity was assessed using I^2^ and Cochran’s Q. Small-study effects were evaluated by visual inspection of the funnel plot and Egger’s regression test, recognizing that both approaches have limited reliability with a modest number of heterogeneous studies.

Prespecified subgroup analyses were performed by: (1) study design (cohort vs case-control); (2) HIV co-infection status of the study population (HIV-specific vs non-HIV-specific); and (3) CAD outcome (MI as the primary endpoint vs broader CAD outcomes). Between-subgroup heterogeneity was assessed using a chi-squared interaction test. The HIV-specific quantitative subgroup contained four studies (Bedimo et al (2010) [28], Campbell et al (2012) [21], the D:A:D Study (2010) [30], and Williams-Nguyen et al (2020) [35]); the study of Enger et al (2014) [16] was classified as non-HIV-specific. Three additional HIV-focused reports (Freiberg et al 2011 [17], Freiberg et al (2013) [25], and Lang et al (2022) [32]) were retained in the qualitative review but were not represented in the original quantitative subgroup; the resulting narrow evidence base is acknowledged in the limitations. Meta-regression examined mean follow-up duration, mean age in the HCV-infected group, and the proportion of male participants. Leave-one-out sensitivity analysis assessed the influence of each study. Analyses and figures were generated using Stata.

### Institutional Review Board approval

Not applicable. This systematic review and meta-analysis used previously published data and did not involve direct enrollment of human participants or access to identifiable individual-level data.

### Ethical compliance with human/animal study

Not applicable. No new human or animal participants were enrolled or directly studied in this systematic review and meta-analysis.

## Results

### Study selection

The systematic search across the four electronic databases yielded 3,421 records. The subsequent articles were evaluated concerning eligibility after the deduplication and title/abstract screening. After the full-text review and exclusion of articles that did not meet the prespecified inclusion criteria, 21 articles were included (Fig. 1).

**Figure 1:**
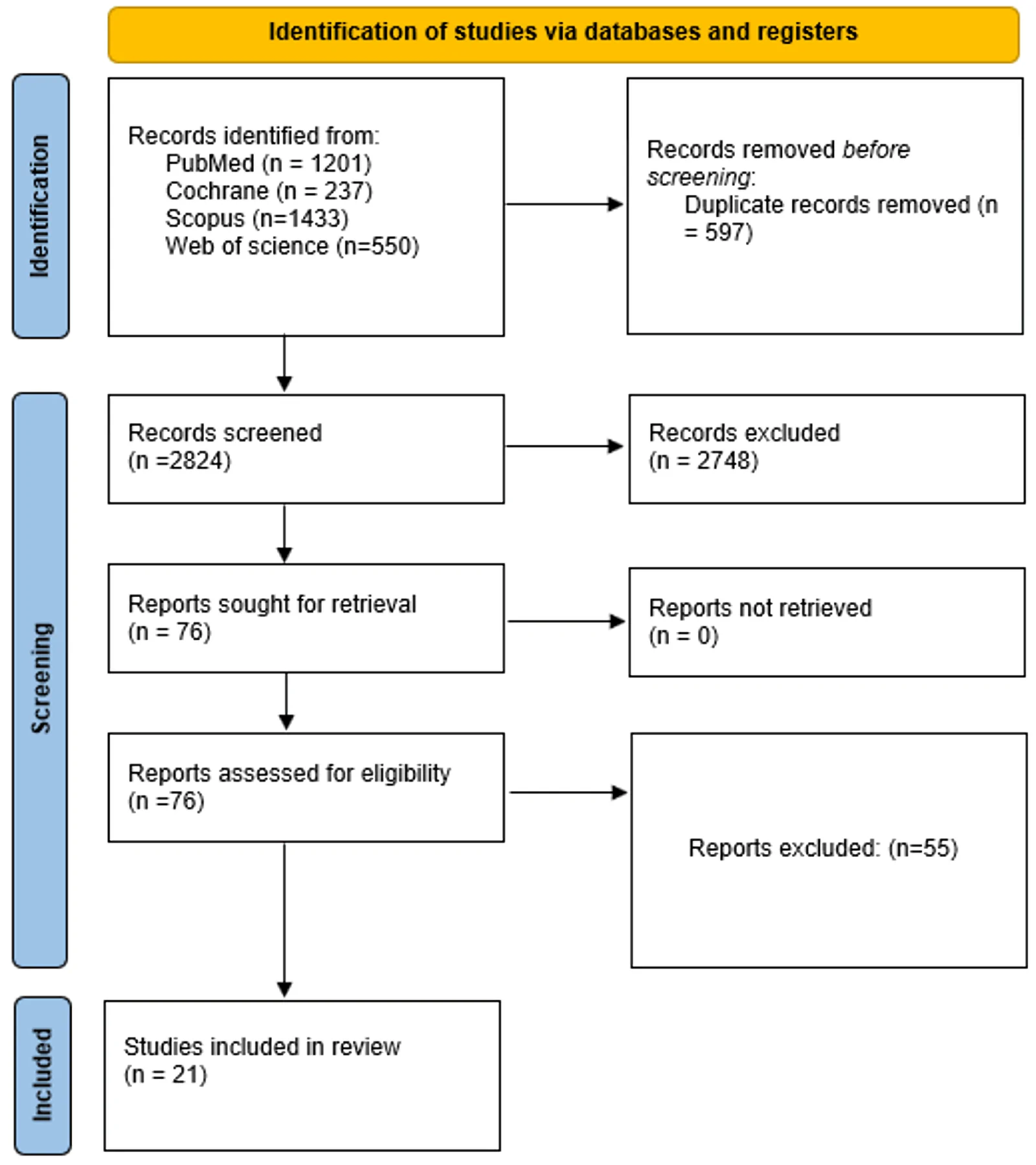
PRISMA flow diagram of study identification, screening, eligibility assessment, and inclusion in the systematic review and meta-analysis. PRISMA: Preferred Reporting Items for Systematic Reviews and Meta-Analyses.

### Study characteristics

The 21 included studies were published in 2004–2023 and included different study designs, including cohort (n = 16), case-control (n = 4), and a single cross-sectional study [16–36]. A combination of serological antibody tests, International Classification of Diseases, Ninth Revision (ICD-9) diagnostic code, and/or HCV RNA quantification was used to diagnose HCV. CAD outcomes were a heterogeneous mix of MI, ACS, incident CAD, CHD, and cardiovascular mortality, all of which were mostly defined by ICD-9 coding or angiographic reports. Some studies examined specific outcomes among HIV-coinfected populations [17, 21, 25, 28, 30, 32, 35]; others conducted them on general veterans, diabetic patients, maintenance hemodialysis patients, or community health participants (Table 1) [16–36].

Mean participant age in HCV-positive groups varied between around 37 years and 66 years [30, 31]. Male participants were typically high, as is the case with the composition of the veteran-based cohorts. Other comorbidities, including hypertension, diabetes mellitus, and dyslipidemia, were often reported, with a large range of prevalence in the studies; detailed baseline characteristics of each study are presented in Table 2 [16–36].

### Risk of bias assessment

Among the 20 reviewed cohort and case-control studies (Supplementary Material 2, cr.elmerpub.com), most of them were rated as being of good quality (n = 14) [16, 17, 19, 20, 22, 25, 26, 28, 30, 33, 36]; other studies were rated as fair quality [21, 24, 27, 29, 31, 32, 35]. This single cross-sectional study was rated as good (Supplementary Material 3, cr.elmerpub.com) [18]. The main sources of bias were related to incomplete control of confounding variables and the lack of long-term follow-up information.

### Association between HCV and CAD

Fifteen studies contributed adjusted estimates to the random-effects model. HCV infection was associated with a modest increase in the summary relative association estimate for CAD (1.21; 95% CI, 1.05–1.40; P = 0.007), with substantial heterogeneity (I^2^ = 72.1%; P < 0.001) (Fig. 2). Visual inspection of the funnel plot suggested asymmetry, although Egger’s regression test was not statistically significant (P = 0.9687) (Fig. 3). Given the limited number of studies and substantial heterogeneity, the nonsignificant Egger test should not be interpreted as evidence that publication bias or small-study effects are absent. Leave-one-out estimates remained statistically significant, but heterogeneity fell to 42.8% when the study of Kalantar-Zadeh et al [29] was omitted (Supplementary Material 4, cr.elmerpub.com), indicating that this hemodialysis cardiovascular-mortality study materially influenced heterogeneity.

**Figure 2:**
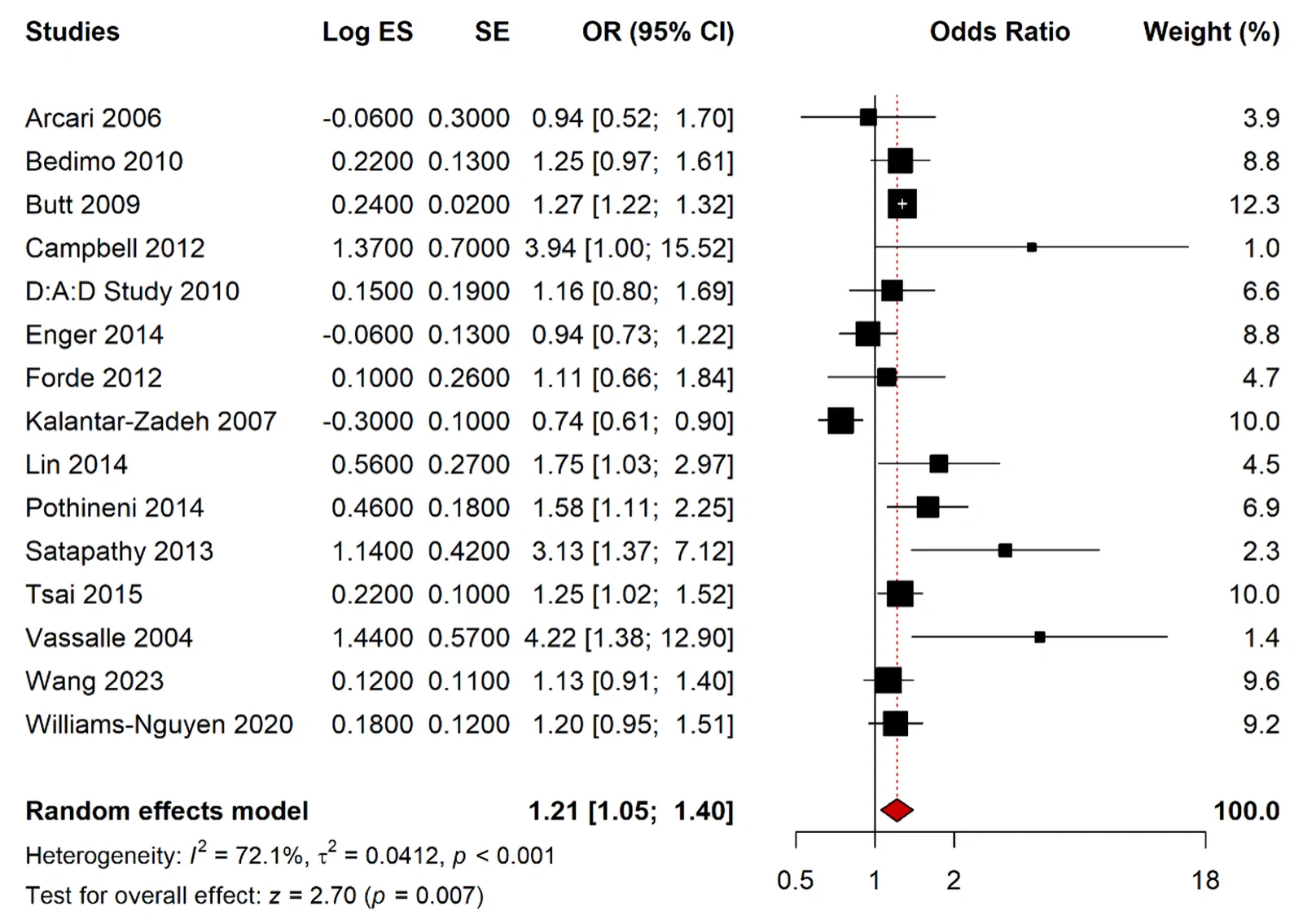
Forest plot of the association between HCV infection and CAD. HCV: hepatitis C virus; CAD: coronary artery disease; CI: confidence interval.

**Figure 3:**
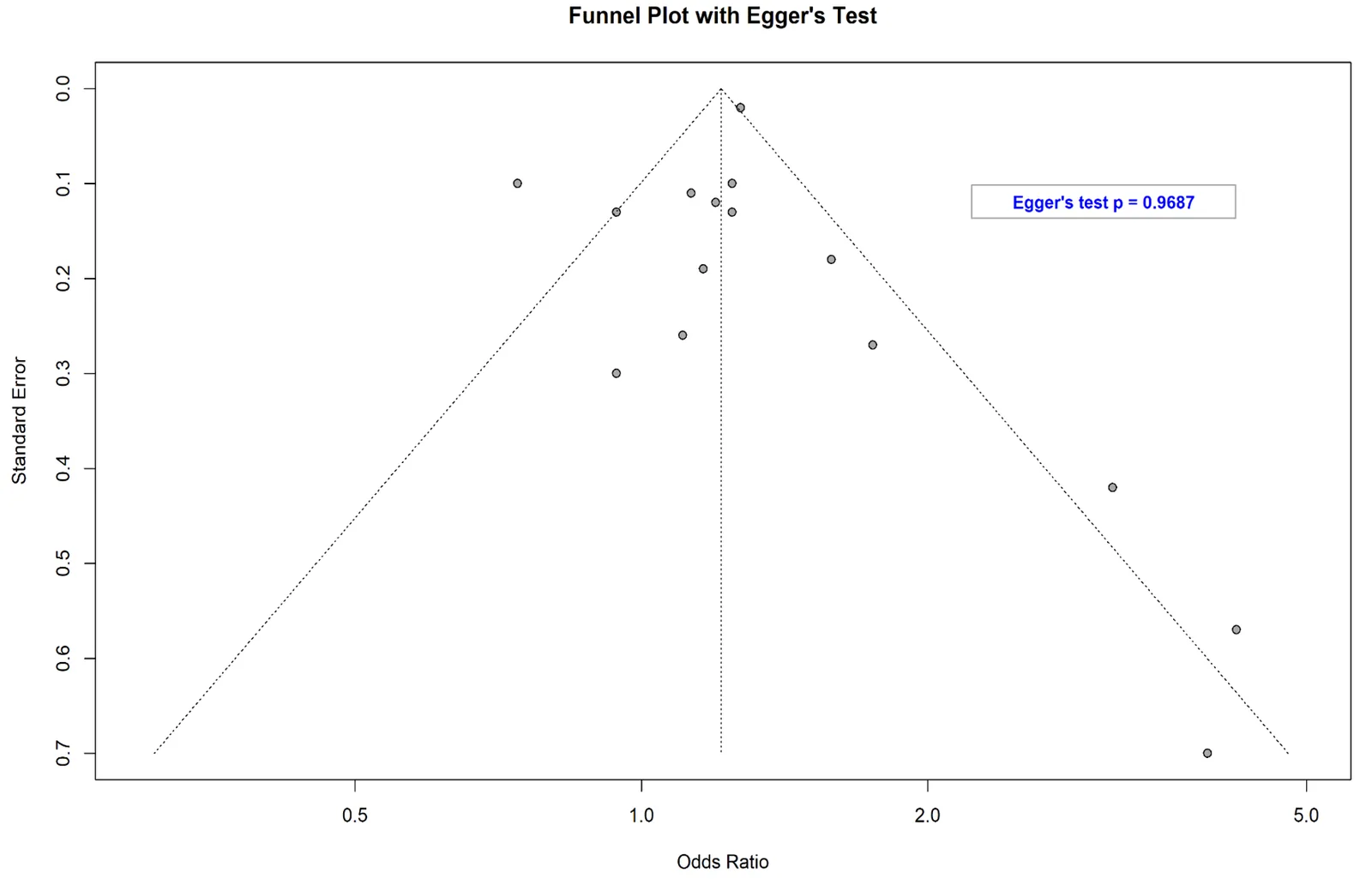
Funnel plot with Egger’s regression test for the association between HCV infection and CAD. HCV: hepatitis C virus; CAD: coronary artery disease.

By study design, cohort studies showed a modest statistically significant association (summary estimate 1.15; 95% CI, 1.00–1.31; P = 0.04; I^2^ = 73.9%), whereas case-control studies did not (2.14; 95% CI, 0.84–5.47; P = 0.11; I^2^ = 76.5%) (Fig. 4). The between-subgroup difference was not statistically significant (P = 0.15).

**Figure 4:**
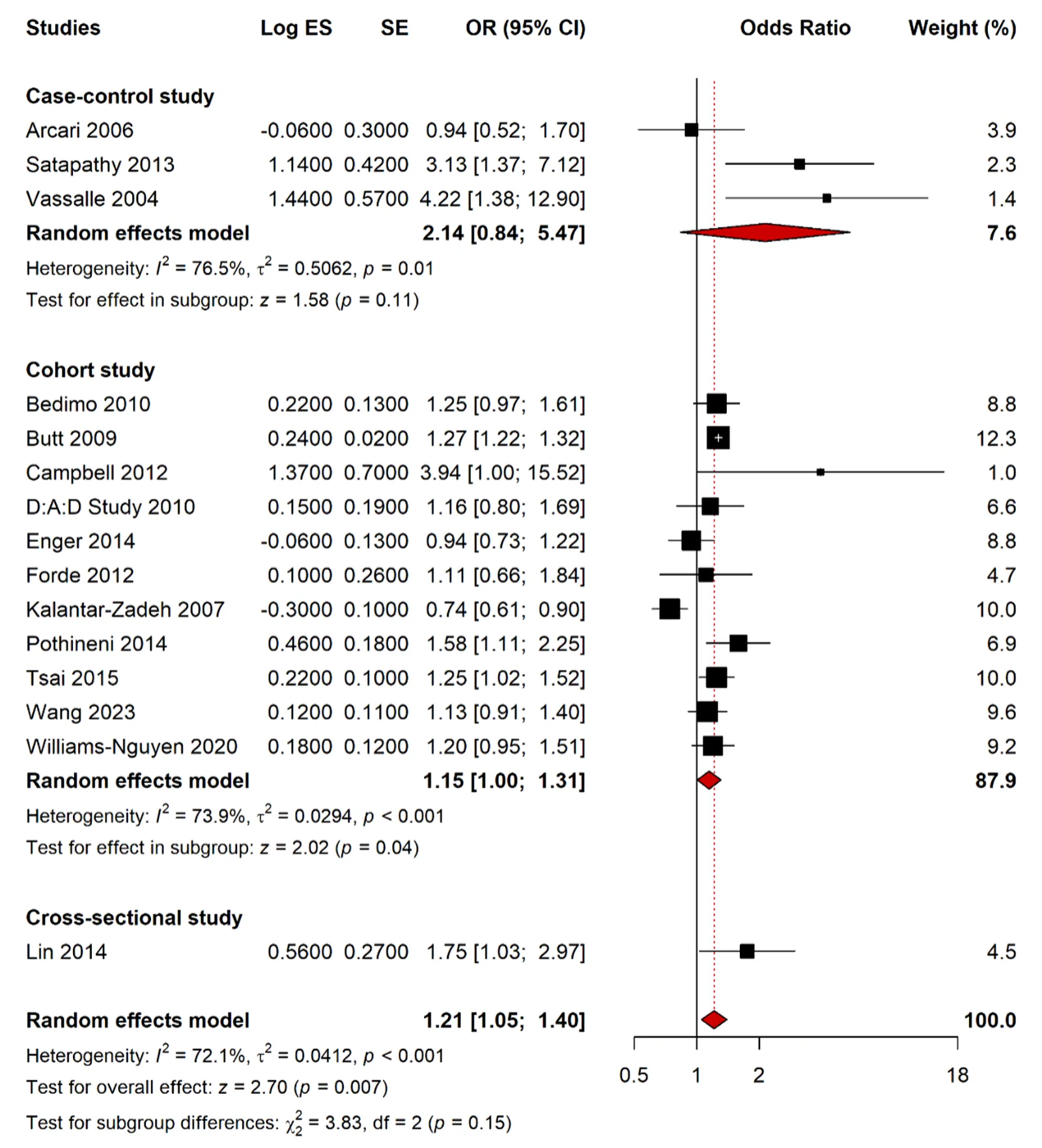
Forest plot of the association between HCV infection and CAD, stratified by study design. HCV: hepatitis C virus; CAD: coronary artery disease; CI: confidence interval.

The HIV-stratified analysis yielded the same point estimate in the non-HIV-specific subgroup (1.23; 95% CI, 1.01–1.49; P = 0.04) and the HIV-specific subgroup (1.23; 95% CI, 1.05–1.43; P = 0.010) (Fig. 5). The non-HIV-specific subgroup had substantial heterogeneity (I^2^ = 78.9%; P < 0.001), whereas no heterogeneity was detected in the four-study HIV-specific subgroup (I^2^ = 0%; P = 0.41). The between-subgroup difference was not statistically significant (P > 0.99). Because the HIV-specific analysis was based on only four studies, these findings should be interpreted as exploratory rather than as evidence of equivalence across HIV strata.

**Figure 5:**
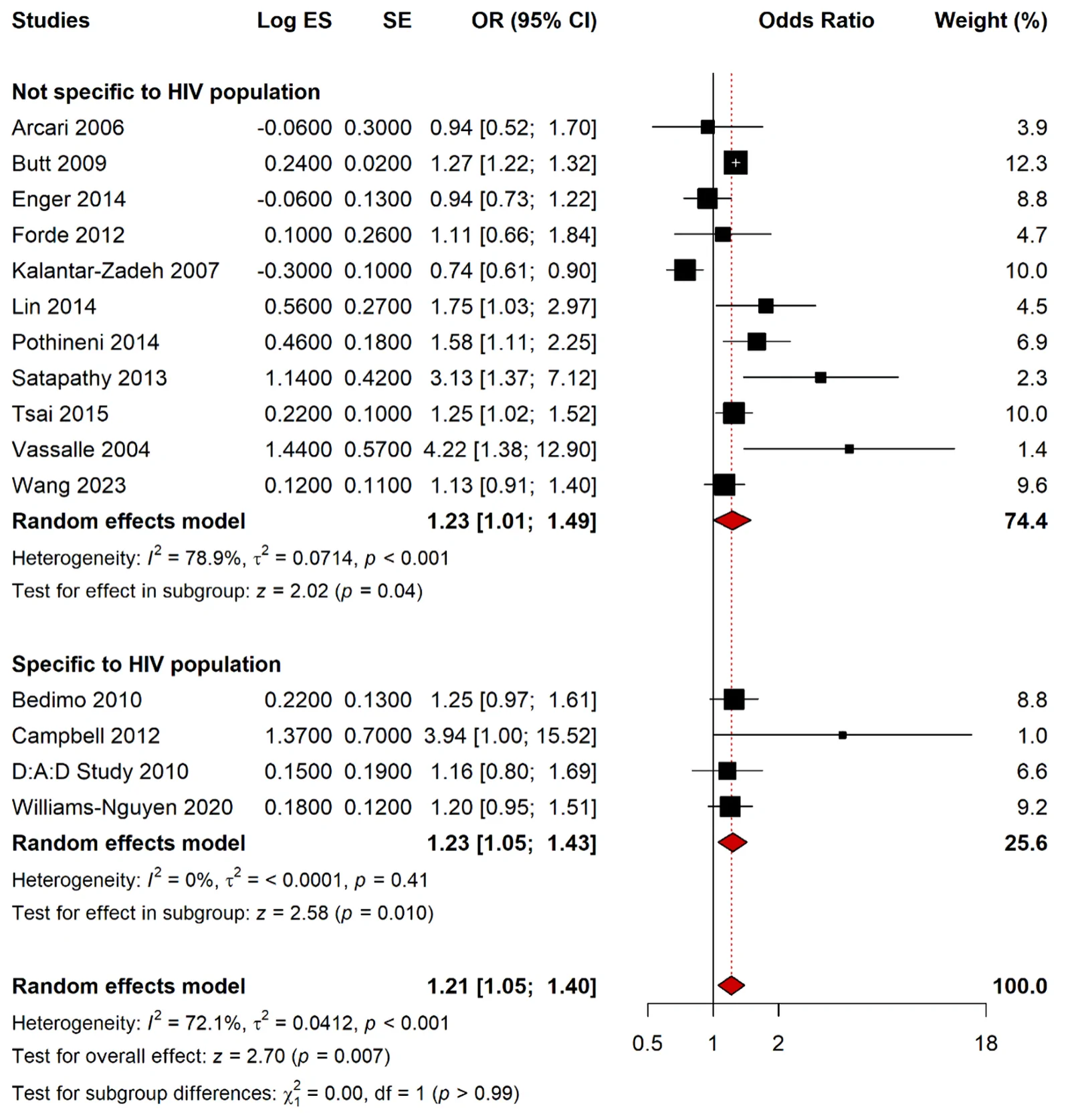
Forest plot of the association between HCV infection and CAD, stratified by HIV status of the study population. HCV: hepatitis C virus; CAD: coronary artery disease; HIV: human immunodeficiency virus; CI: confidence interval.

Results differed by CAD endpoint (Fig. 6). In studies using MI as the primary endpoint, the pooled estimate was not statistically significant (1.12; 95% CI, 0.99–1.27; P = 0.08; I^2^ = 0%; P for heterogeneity = 0.68). In studies using broader or non-specified CAD endpoints, the pooled estimate was significant (1.44; 95% CI, 1.07–1.93; P = 0.02) with substantial heterogeneity (I^2^ = 81.9%; P < 0.001). The between-subgroup difference was not statistically significant (P = 0.12).

**Figure 6:**
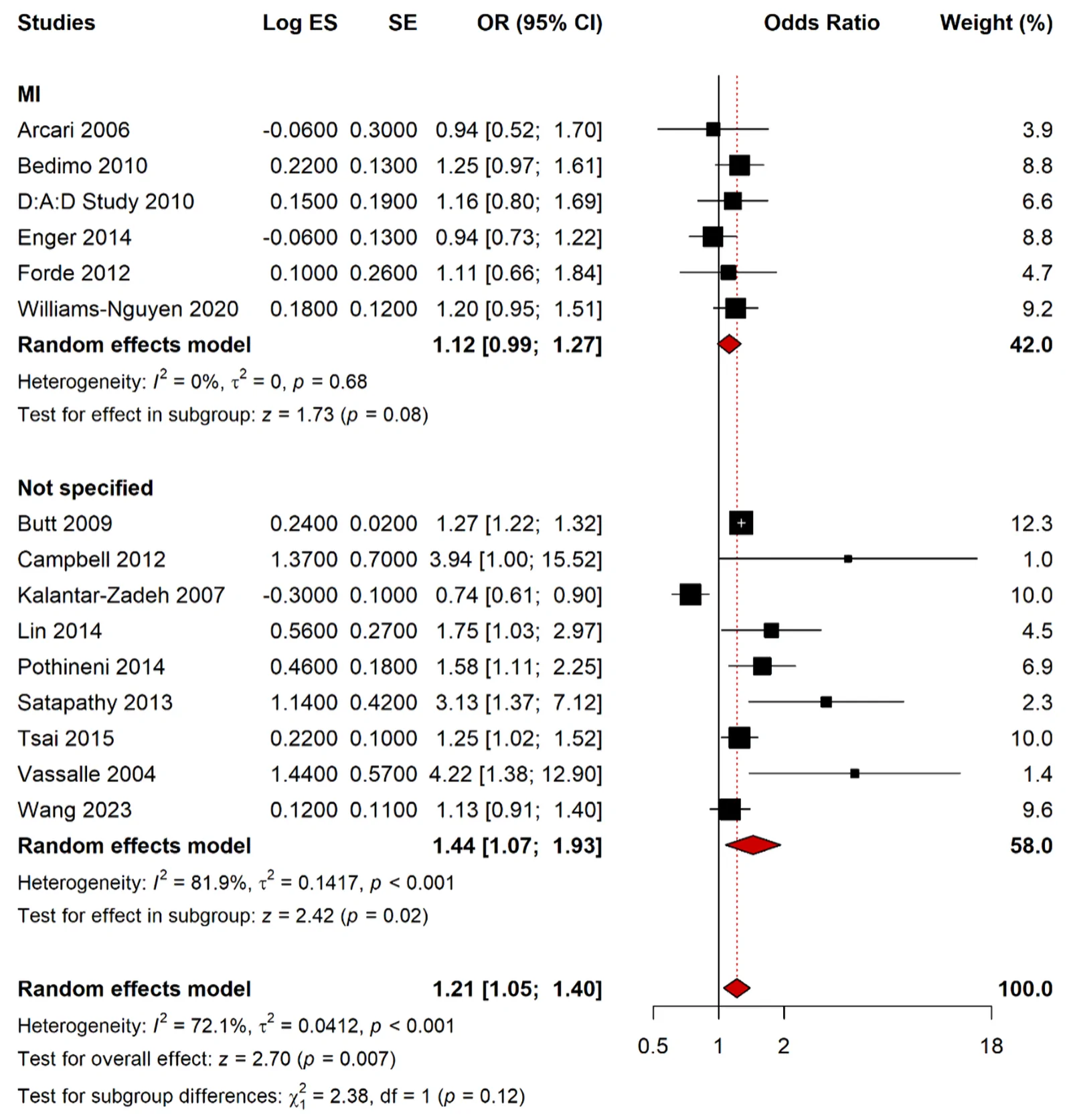
Forest plot of the association between HCV infection and CAD, stratified by CAD outcome type. HCV: hepatitis C virus; CAD: coronary artery disease; MI: myocardial infarction; CI: confidence interval.

Meta-regression did not identify statistically significant associations with mean follow-up duration (P = 0.15), mean age (P = 0.342), or the proportion of male participants (P = 0.92) (Fig. 7; Supplementary Materials 5, 6, cr.elmerpub.com). These study-level analyses were exploratory and may have been underpowered.

**Figure 7:**
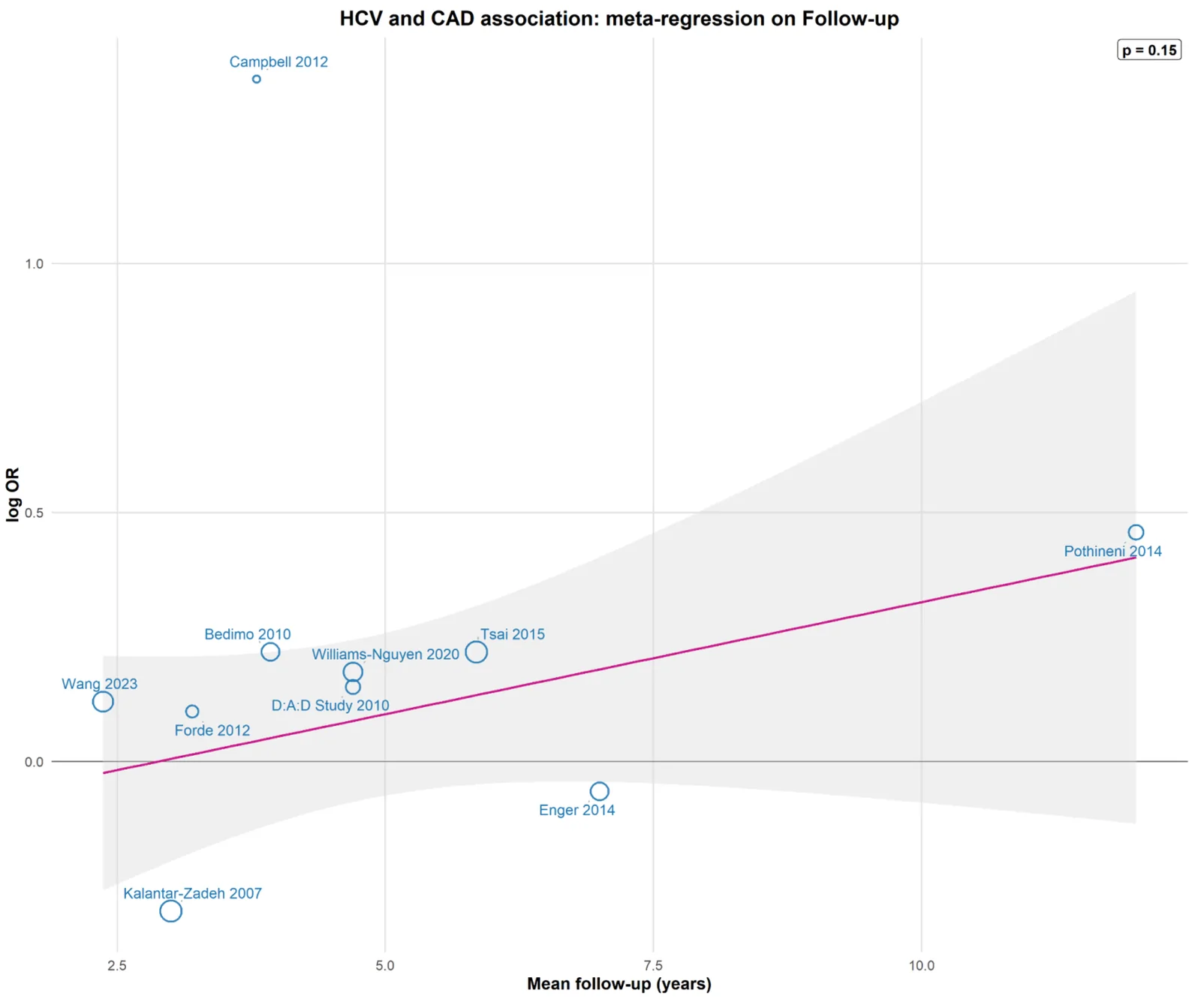
Meta-regression of follow-up duration for the association between HCV infection and CAD. HCV: hepatitis C virus; CAD: coronary artery disease.

## Discussion

This meta-analysis found a modest positive association between chronic HCV infection and CAD (summary estimate 1.21), but the interpretation is constrained by substantial between-study heterogeneity and by the combination of clinically distinct outcomes and effect measures. The association was present in cohort studies and in both HIV-stratified subgroups, whereas the MI-specific subgroup did not reach statistical significance. Accordingly, the findings support a small epidemiological association but do not establish that HCV is an independent causal risk factor or that the association has clear clinical importance for an individual patient.

Several mechanisms could plausibly contribute to an association, including systemic inflammation, endothelial dysfunction, oxidative stress, and HCV-related steatosis and insulin resistance [6–9]. At the same time, active HCV infection is often associated with lower LDL cholesterol, which would ordinarily be expected to attenuate atherosclerotic risk [10, 11]. These countervailing biological features reinforce that mechanistic plausibility alone cannot establish causality and that treatment era, comorbidity, and residual confounding must be considered.

The contrast between MI-specific and broader CAD outcomes may reflect endpoint heterogeneity rather than a true mechanistic difference. Adjudicated incident MI, ICD-coded ACS/CAD, ischemic electrocardiographic findings, revascularization, and cardiovascular mortality are not clinically interchangeable. This concern is supported by the leave-one-out analysis: removing the study of Kalantar-Zadeh et al [29], a hemodialysis cohort with cardiovascular mortality rather than incident CAD as the endpoint, reduced I^2^ from 72.1% to 42.8% [29]. The primary pooled estimate is therefore best interpreted as an average association across heterogeneous cardiovascular definitions, not as a precise estimate for incident MI.

The overall direction and magnitude of the association are broadly consistent with prior syntheses rather than uniquely novel. Ambrosino et al reported a CAD estimate of 1.43 [12]. Petta et al separately evaluated cardiovascular mortality, carotid atherosclerosis, and cerebrocardiovascular events in 22 studies [13]. Lee et al included 36 longitudinal studies and reported a pooled RR of 1.28 (95% CI, 1.18–1.39), with separate analyses for composite cardiovascular events, MI, and stroke [14]. The present review contributes an updated CAD-focused dataset, HIV-stratified exploratory analyses, and study-level meta-regression, but it should not be interpreted as the first outcome-stratified synthesis or as superseding the larger analysis by Lee et al [14].

The HIV-stratified findings also require caution. Only four studies contributed to the HIV-specific quantitative subgroup, although three additional HIV-focused reports (Freiberg et al (2011) [17], Freiberg et al (2013) [25], and Lang et al (2022) [32]) were included in the qualitative review. The identical subgroup point estimates and nonsignificant interaction test do not establish equivalence or independence from HIV status. The subgroup is better viewed as hypothesis-generating pending a prospectively specified analysis with complete, non-overlapping study-level estimates.

### Strengths

Strengths include a comprehensive four-database search without date or language restrictions, duplicate screening and extraction, use of adjusted estimates, and prespecified subgroup and meta-regression analyses. Based on reported study totals, the 15 quantitatively pooled studies represented approximately 726,784 participant records, and all 21 included reports represented approximately 1.27 million participant records. Because several reports drew from large administrative or veteran cohorts and may contain overlapping source populations, these aggregates should not be interpreted as numbers of unique individuals.

### Limitations

First, all included studies were observational, so residual confounding remains likely. Renal disease is particularly important because chronic kidney disease and end-stage renal disease strongly influence CAD and cardiovascular mortality, yet renal function was inconsistently measured or adjusted across studies. The influence of the Kalantar-Zadeh et al [29] hemodialysis cohort underscores this concern. Other incompletely captured factors included HCV genotype and viral load, cirrhosis severity, smoking and substance use, socioeconomic factors, HIV-related characteristics, and baseline cardiovascular risk.

Second, antiviral exposure varied across treatment eras and was not sufficiently reported to distinguish untreated infection, interferon-based therapy, and direct-acting antiviral regimens or to examine specific agents, treatment duration, sustained virologic response, or time since cure. Lipid-lowering therapy and other cardioprotective treatments were also inconsistently reported between HCV groups and could have modified outcomes. Consequently, this review cannot estimate the cardiovascular effect of antiviral therapy or lipid-lowering treatment.

Third, HRs, RRs, and ORs were pooled as log-relative measures despite differences in their mathematical interpretation, baseline event risks, follow-up duration, and covariate adjustment. The rare-outcome assumption could not be verified because cumulative incidence and absolute event rates were inconsistently reported, which is particularly relevant to cardiovascular mortality in end-stage renal disease. Adjusted ORs are also non-collapsible, and the materially different covariate sets shown in Table 1 [16–36] further limit comparability. The aggregate information and original analysis files available for this revision were insufficient to perform reliable effect-measure-specific sensitivity analyses, a new primary analysis excluding Kalantar-Zadeh et al [29], or a *post hoc* expansion of the HIV subgroup while resolving non-equivalent comparators and potential cohort overlap. These unavailable analyses are therefore acknowledged as limitations rather than replaced by unverified estimates.

Fourth, outcome definitions and HCV ascertainment varied, many outcomes were identified from ICD codes, and substantial residual heterogeneity remained unexplained. The population was dominated by male US veteran cohorts, limiting generalizability to women and non-Western populations; sex-related differences may influence cardiovascular outcomes [37–39]. The visually asymmetric funnel plot raises concern for small-study or publication effects despite a nonsignificant Egger test, which has limited power in this setting.

### Conclusions

Chronic HCV infection was associated with a modest increase in the pooled relative estimate for CAD, but the clinical significance is uncertain. Substantial heterogeneity, non-equivalent endpoints and populations, residual confounding, and effect-measure limitations prevent causal interpretation. The nonsignificant MI-specific analysis and the sensitivity of heterogeneity to a hemodialysis cardiovascular-mortality study further support caution. These data do not demonstrate that antiviral therapy reduces CAD. Future prospective studies should use standardized incident CAD and MI endpoints and report renal function, HCV treatment regimen and virologic response, lipid-lowering therapy, and absolute event rates.

## Supplementary Material

Suppl 1 Search strategy across databases.

Suppl 2 Risk of bias assessment for cohort and case-control studies using the NOS tool.

Suppl 3 Risk of bias assessment of cross-sectional studies using the NOS tool.

Suppl 4 Leave-one-out meta-analysis for the association between HCV and CAD.

Suppl 5 Meta-regression on age for the association between HCV and CAD.

Suppl 6 Meta-regression on male gender for the association between HCV and CAD.

## Data Availability

Any inquiries regarding supporting data availability of this study should be directed to the corresponding author.
