# Supplementary material for: Association Between Hepatitis C Virus Infection and Coronary Artery Disease: A Systematic Review and Meta-Analysis
Source: Cardiol Res. 2026 Oct 24;17(6):670–89. doi: 10.14740/cr2280 (PMC13719658; doi:10.14740/cr2280)
Supplement: Supplementary file 1 [file cr-17-06-670-s001.docx]

**Suppl 1.** Search strategy across databases.

| **Database** | **Search term** | **Filters** | **Results** |
| --- | --- | --- | --- |
| PubMed | (“hepatitis C” OR “hepatitis C virus” OR HCV) AND ("cardiovascular disease" OR CVD OR "coronary artery disease" OR CAD OR "coronary heart disease" OR CHD OR "ischemic heart disease" OR IHD OR "myocardial infarction" OR MI OR "heart failure" OR "congestive heart failure" OR CHF OR "acute coronary syndrome" OR ACS OR "unstable angina" OR UA OR angina) | Title-abstract | 1201 |
| Scopus | (“hepatitis C” OR “hepatitis C virus” OR HCV) AND ("cardiovascular disease" OR CVD OR "coronary artery disease" OR CAD OR "coronary heart disease" OR CHD OR "ischemic heart disease" OR IHD OR "myocardial infarction" OR MI OR "heart failure" OR "congestive heart failure" OR CHF OR "acute coronary syndrome" OR ACS OR "unstable angina" OR UA OR angina) AND (cohort OR "case-control" OR "case control" OR "case-cohort" OR "case cohort" OR "self-controlled case series" OR "self controlled case series" OR longitudinal OR observational) | Title-abstract | 1433 |
| WOS | (“hepatitis C” OR “hepatitis C virus” OR HCV) AND ("cardiovascular disease" OR CVD OR "coronary artery disease" OR CAD OR "coronary heart disease" OR CHD OR "ischemic heart disease" OR IHD OR "myocardial infarction" OR MI OR "heart failure" OR "congestive heart failure" OR CHF OR "acute coronary syndrome" OR ACS OR "unstable angina" OR UA OR angina) AND (cohort OR "case-control" OR "case control" OR "case-cohort" OR "case cohort" OR "self-controlled case series" OR "self controlled case series" OR longitudinal OR observational) | Topic | 550 |
| Cochrane | (“hepatitis C” OR “hepatitis C virus” OR HCV) AND ("cardiovascular disease" OR CVD OR "coronary artery disease" OR CAD OR "coronary heart disease" OR CHD OR "ischemic heart disease" OR IHD OR "myocardial infarction" OR MI OR "heart failure" OR "congestive heart failure" OR CHF OR "acute coronary syndrome" OR ACS OR "unstable angina" OR UA OR angina) | Title-abstract | 237 |
