# Supplementary material for: Association Between Hepatitis C Virus Infection and Coronary Artery Disease: A Systematic Review and Meta-Analysis
Source: Cardiol Res. 2026 Oct 24;17(6):670–89. doi: 10.14740/cr2280 (PMC13719658; doi:10.14740/cr2280)
Supplement: Supplementary file 2 [file cr-17-06-670-s002.docx]

**Suppl 2.** Risk of bias assessment for cohort and case-control studies using the Newcastle-Ottawa Scale (NOS) tool.

| **Study ID** | **Type of study** | **Selection** | | | | **Comparability** | **Outcome** | | | **Overall quality** |
| --- | --- | --- | --- | --- | --- | --- | --- | --- | --- | --- |
|  |  | **Is the case definition adequate/ Representativeness of the exposed cohort** | **Representativeness of the cases/ Selection of the non-exposed cohort** | **Selection of Controls/ Ascertainment of exposure** | **Definition of Controls/ Demonstration that the outcome of interest was not present at the start of the study** | **Comparability of cases and controls (cohorts) on the basis of the design or analysis** | **Ascertainment of exposure/ Assessment of outcome** | **Same method of ascertainment for cases and controls/ Was follow-up long enough for outcomes to occur** | **Non-Response rate/ Adequacy of follow up of cohorts** |  |
| **Arcari 2006** | Case-control study | * | * | * | * | * | * | * |  | Good quality |
| **Bedimo 2010** | Cohort study | * | * | * | * |  | * | * | * | Good quality |
| **Butt 2007** | Cohort study | * | * | * | * | * | * | * |  | Good quality |
| **Butt 2009** | Cohort study | * | * | * | * |  | * | * |  | Fair quality |
| **Butt 2017** | Cohort study | * | * | * | * |  | * | * | * | Good quality |
| **Campbell 2012** | Cohort study | * | * | * | * |  | * | * |  | Fair quality |
| **DAD study 2010** | Cohort study | * | * | * | * | * | * | * |  | Good quality |
| **Enger 2014** | Cohort study | * | * | * | * | * | * | * | * | Good quality |
| **Forde 2012** | Cohort study | * | * | * | * | * | * | * | * | Good quality |
| **Freiberg 2011** | Cohort study | * | * | * | * | * | * | * |  | Good quality |
| **Freiberg 2013** | Cohort study | * | * | * | * | * | * | * |  | Good quality |
| **Hsu 2014** | Cohort study | * | * | * | * | * | * | * |  | Good quality |
| **Kalantar-Zadeh 2007** | Cohort study | * | * | * | * |  | * | * |  | Fair quality |
| **Lang 2022** | Cohort study | * | * | * | * |  | * | * |  | Fair quality |
| **Pothineni 2014** | Cohort study | * | * | * | * |  | * | * |  | Fair quality |
| **Satapathy 2013** | Case-control study | * | * | * | * | ** | * | * | * | Good quality |
| **Tsai 2015** | Cohort study | * | * | * | * | * | * | * | * | Good quality |
| **Vassalle 2004** | Case-control study | * | * | * | * |  | * | * |  | Fair quality |
| **Wang 2023** | Cohort study | * | * | * | * | ** | * | * | * | Good quality |
| **Williams-Nguyen 2020** | Cohort study | * | * | * | * |  | * | * |  | Fair quality |
