# Supplementary material for: Association Between Hepatitis C Virus Infection and Coronary Artery Disease: A Systematic Review and Meta-Analysis
Source: Cardiol Res. 2026 Oct 24;17(6):670–89. doi: 10.14740/cr2280 (PMC13719658; doi:10.14740/cr2280)
Supplement: Supplementary file 3 [file cr-17-06-670-s003.docx]

**Suppl 3.** Risk of bias assessment of cross-sectional studies using the NOS tool.

| **Study ID** | **Selection** | | | | **Comparability** | **Outcome** | | **Quality** |
| --- | --- | --- | --- | --- | --- | --- | --- | --- |
|  | **Representativeness of the sample** | **Sample size** | **Non-respondents** | **Ascertainment of the exposure (risk factor):** | **The subjects in different outcome groups are comparable, based on the study design  or analysis. Confounding factors are controlled** | **Assessment of the outcome** | **Statistical test** |  |
| **Lin 2014** | * | * |  | ** |  | ** | * | Good quality |
