# Supplementary material for: Association Between Hepatitis C Virus Infection and Coronary Artery Disease: A Systematic Review and Meta-Analysis
Source: Cardiol Res. 2026 Oct 24;17(6):670–89. doi: 10.14740/cr2280 (PMC13719658; doi:10.14740/cr2280)
Supplement: Supplementary file 6 [file cr-17-06-670-s006.docx]

**Suppl 6.** Meta-regression on male gender for the association between HCV and CAD.
